# Gas Sensors Based on Porous Ceramic Bodies of MSnO_3_ Perovskites (M = Ba, Ca, Zn): Formation and Sensing Properties towards Ethanol, Acetone, and Toluene Vapours

**DOI:** 10.3390/molecules27092889

**Published:** 2022-04-30

**Authors:** Yasser H. Ochoa-Muñoz, Ruby Mejía de Gutiérrez, Jorge E. Rodríguez-Páez, Isabel Gràcia, Stella Vallejos

**Affiliations:** 1 Composites Materials Group (GMC-CENM), Universidad del Valle, Cali 76001, Colombia; 2 CYTEMAC Group, Universidad del Cauca, Popayán 190003, Colombia; jnpaez@unicauca.edu.co; 3 Institute of Microelectronics of Barcelona (IMB-CNM, CSIC), 08193 Bellaterra, Spain; isabel.gracia@imb-cnm.csic.es (I.G.); stella.vallejos@imb-cnm.csic.es (S.V.)

**Keywords:** gas sensors, perovskites, porous ceramic, ZnSnO_3_, BaSnO_3_, CaSnO_3_

## Abstract

In this work, the gas-sensing functionality of porous ceramic bodies formed by the slip casting technique was studied using perovskite nanoparticles of an MSnO_3_ system (M = Ba, Ca, Zn) synthesized by a chemical route. The performance and reliability of the sensitive materials in the presence of different volatile organic compounds (acetone, ethanol, and toluene), and other gases (CO, H_2_ and NO_2_) were analysed. The ZnSnO_3_, BaSnO_3_, and CaSnO_3_ sensors showed sensitivities of 40, 16, and 8% ppm^−1^ towards acetone, ethanol, and toluene vapours, respectively. Good repeatability and selectivity were also observed for these gaseous analytes, as well as excellent stability for a period of 120 days. The shortest response times were recorded for the ZnSnO_3_ sensors (e.g., 4 s for 80 ppm acetone) with marked responses to low concentrations of acetone (1000 ppb). These results are attributed to the porosity of the sensitive materials, which favours the diffusion of gases, induces surface defects, and provides greater surface area and good sensitivity to acetone, as is seen in the case of ZnSnO_3_.

## 1. Introduction

Metal oxide (MOx) gas sensors are of high interest due to their small size, low cost [1], and rapid response compared with traditional instruments, such as mass spectrometry and gas chromatography [2], hence the interest in investigating, in a more systematic and rigorous way, the sensitivity, selectivity, and stability of these materials in the presence of a particular gas. This type of sensor can be used in environmental protection [3], safety instruments in laboratories and industry [4], and in the field of medicine for the early diagnosis of diseases [5], among other applications. MOx micro/nanostructures, e.g., based on TiO_2_, ZnO and SnO_2_, have been used as gas-sensitive materials for several decades due to their unique physicochemical properties. However, the lack of selectivity and high operating temperatures (200–500 °C) of these oxides [6] have led to the development of special MOx to further promote the physicochemical properties and thus better expand the practical applications of these materials. The MOx improvements include their doping/decoration with noble metals (e.g., Pd, Ag, Pt, and Au) [7,8], surface functionalization [9,10], and formation of composites (e.g., MOx-MOx, polymer-MOx, and MOx-carbon nanotubes (CNTs)) [11]. These MOx modifications have shown that the detection performance of a gas is mainly mediated by the properties of the surface of the sensitive material and its combination with multiple components that act synergistically to increase the sensitivity, selectivity, and response rates during gas detection. Other factors to consider are morphology and size since a high surface area and a small particle size can improve detection performance, as well as the control of the porosity of the sensitive material through the shaping process.

The use of ternary oxides as gas detectors is also a broad field of research on improving the properties of resistive gas sensors. Among these, the perovskite structure stands out, with a general formula of ABO_3_, where cation B with a smaller ionic radius is placed inside the corner-sharing octahedra BO_6_ that form a three-dimensional network with suitable interstices for cation A [12]. Distortions in the structure of these perovskites normally occur because of subtle changes in the size of the different interstitial sites, as well as the different A and B cations that are used, which have different sizes and valences. These structural alterations generate cationic vacancies since they are much less mobile than oxygen vacancies, which together with electrons and holes determine the electrical properties of perovskites [13]. Therefore, they influence the gas detection properties of the material. 

Perovskite-type oxides have numerous applications as a result of their diverse compositions and structures, excellent thermal stability, redox behaviour, oxygen mobility, and electronic and ionic conductivity [14]. In the last decade, the MOx of the MSnO_3_ system (M = Ba, Ca, Zn) have stood out, with use as dielectric materials [15,16,17], solar cells [18,19], anodes for lithium-ion batteries [20,21,22], and detectors of different gaseous species [23,24,25]. The variation in the interstitial cation M with the elements Ba, Ca, and Zn modifies the capacity to host cationic vacancies and oxygen, giving these stannates variable electrical and redox properties and synergistic catalytic functions, with critical impacts on the gas detection properties.

Research carried out with ZnSnO_3_ has shown that this material has important sensing capacities for different gases [26] including acetone [27], ethanol [28,29,30], formaldehyde [26,31], CO [32], NO_2_ [33], and H_2_ [34]. This does not rule out the possibility of studying the use of other perovskites such as BaSnO_3_ and CaSnO_3_ in gas sensors [25,35,36], considering their technological potential. Previously, to evaluate the sensing capacity of these perovskites, stannate powders synthesized directly or coated as a thin film were used [33,34], but it is not easy to find evaluation reports on ceramic bodies formed from these ceramic powders. Therefore, it is of interest to determine the electrical behaviour of the sintered bodies of these perovskites in the presence of certain gases. Although there are few reports on the formation of these ceramic bodies, several of them deal specifically with sintering and [37,38,39,40,41] have been patented [42,43]. This indicates that there is technological interest in the formation of ceramic bodies using powders of different stannates, an important stage in this work. Generally, the use of sintered bodies of classical tin or zinc oxide has shown advantages over thick films in gas sensing due to a higher control of the grain boundary and the reproducibility of the gas-sensitive material through the sintering temperature and duration of the process.

The colloidal process is a widely used method for obtaining advanced ceramic bodies. This processing technique integrates several ceramic forming techniques, including tape casting, dip coating, screen printing, and slip casting [44,45]. In colloidal processing methods, stable slip is initially formed with ceramic powders of interest in a liquid medium. With this slip, ceramic products with very good characteristics can be formed, considering various applications, among which the following stand out: biosensors, gas sensors, varistors, condensers and fuel cell films [46,47,48,49,50].

Generally, comparative studies of porous sintered bodies or porous, thick films as gas sensors, mainly in classical tin or zinc oxide sensors, have found that the formation of necks between the particles, due to the sintering of the particles, favours the electrical conduction mechanisms. Therefore, sintered bodies may show enhanced electrical response in the presence of gases compared to thick films derived from powder pastes [51,52]. Other prior works also highlighted this fact [53,54,55]. 

In this work, a simplified forming method was developed without complex instrumentation requirements (e.g., screen printers or vacuum chambers) and with repeatable characteristics in terms of the performance of the sensitive materials. This method, slip casting, allowed the formation of small disc-shaped bodies from concentrated suspensions of nanoparticles of the MSnO_3_ system (M = Ba, Ca, Zn). The shaped discs were sintered to improve their densification and mechanical properties while preserving their stoichiometry. In the sintered bodies, the structure, morphology, chemical compositions, and gas-sensing properties were studied. The experimental results show that the bodies of MSnO_3_ (M = Ba, Ca, Zn) exhibit excellent gas-sensing performance towards ethanol, acetone, and toluene vapours. The lowest measurements for these gaseous analytes in humid environments (10–30% relative humidity) were performed at 1000 ppb.

## 2. Materials and Experimental Methodology

### 2.1. Raw Material

The preparation of the compounds and their corresponding structural analysis can be found in [56]. Specifically, particles with sizes between 20 and 100 nm of the MSnO_3_ system (M = Ba, Ca, Zn) synthesized by the complex polymeric method were used. The surface areas of these particles were determined using a surface area and pore analyser Quantachrome NOVA 1000e; the results were 38,913 m^2^·g^−1^, 4056 m^2^·g^−1^, and 2560 m^2^·g^−1^ for BaSnO_3_, CaSnO_3_, and ZnSnO_3_, respectively. 

### 2.2. Sensor Fabrication

**Figure 1:**
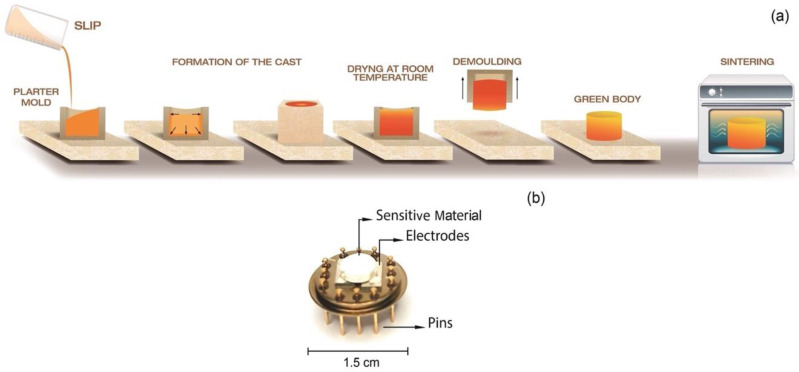
(**a**) Scheme of the procedure carried out for shaping the gas-sensitive material. (**b**) Photograph of a sensor containing the sensitive material and the electrodes integrated in a TO-8 package.

To form the gas-sensitive material (a disc-shaped ceramic body), the slip casting technique was used. This method includes the preparation of aqueous suspensions (slurries) with the ceramic powders of interest. The proportions of distilled water and solid content (i.e., MSnO_3_ perovskites (M = Ba, Ca, Zn)) were experimentally determined by viscosity (ɳ) vs. solid content (φ) curves, using the methodology for the preparation of colloidal suspensions and the testing of their rheological properties in [57]. The rheological tests were performed using a Haake Mars III rheometer from Thermo Scientific with a concentric cylinder configuration. The optimal solid contents of the suspensions were determined to be 13.7, 19.2, and 21.3 vol% for the BaSnO_3_, ZnSnO_3_, and CaSnO_3_ systems, respectively. Subsequently, the slurries were poured into plaster-type disc-shaped moulds (70 mm in diameter and 30 mm in thickness). The liquid was sucked into the areas in contact with the walls of the mould, forming a layer of compact packed particles, which grew in the suspension from the walls of the mould. The compact material formed was removed from the mould and left in a desiccator for 24 h. A diagram of the methodology used to obtain the green body of the sensitive material, previously described, is presented in Figure 1a. 

The ceramic specimens were sintered in a Carbolite RHF 1600 electric furnace at temperatures between 1000–1500 °C for 1 h. A helium pycnometer (Ultrapyc gas pycnometer 3000, Anton-Paar, Graz, Austria) was used to determine the density in the sintered bodies. The apparent porosity (*AP*%) was calculated using Equation (1), described in the ASTM Standard C20 and in [58].

(1) $$AP\%=\left[\frac{W-D}{W-S}\right]\times 100\%$$

where *D* is the weight in air (g); *W* is the soaked weight (g); and *S* is the suspended weight (g). The apparent porosity expresses as a percentage the relationship of the volume of the open pores in the specimen to its exterior volume. 

Their structure, chemical composition, morphology, and pore size distribution were studied by X-ray diffraction (XRD—Bruker, AXS D8 Advance operated at 40 kV and 40 mA, Cu Kα radiation, Karlsruhe, Germany), Raman spectroscopy (Raman—Horiba XploRa, Kyoto, Japan), X-ray photoelectron spectroscopy (XPS—Kratos Axis Supra spectrometer, with Al/Ag monochromatic X-ray source, Manchester, UK), scanning electron microscopy (SEM—Carl Zeiss, Auriga Series, Oberkochen, Germany), and mercury porosimetry (Micromeritics, AutoPore IV 9500 model, Norcross, GA, USA), respectively. The XPS spectra were deconvoluted with CasaXPS software version 2.3.24 (Computer aided surface analysis for X-Ray photoelectron spectroscopy, Casa Software Ltd., Devon, UK) using a Gaussian/Lorentzian (85/15) product function after subtraction of a Shirley nonlinear sigmoid-type baseline. The sensitive materials (sintered ceramic bodies) were manually integrated using silver paint to improve adhesion on the platinum electrodes that make up the system. The integrated sensor is shown in Figure 1b.

### 2.3. Sensor Testing

The sensors based on sintered bodies were tested using a system equipped with 6 chambers connected in parallel (each with a volume of approximately 3.2 mL) with continuous flow, equipped with mass flow controllers that allowed a mixture of dry/humid air. Calibrated analyte gases (acetone, ethanol, toluene, CO, H_2_, and NO_2_ purchased from Praxair, Swindon, UK) were used to obtain the desired concentration. Moisture was generated by bubbling, and the relative humidity (RH) control inside the gas chamber was monitored with an evaluation kit (EK-H4, Sensirion AG, Stäfa, Switzerland) equipped with a humidity sensor. An adjustable heating plate (Combiplac, JP SELECTA, Barcelon, Spain) was used to set the temperature of the sensitive material, which was monitored during the test. 

**Figure 2:**
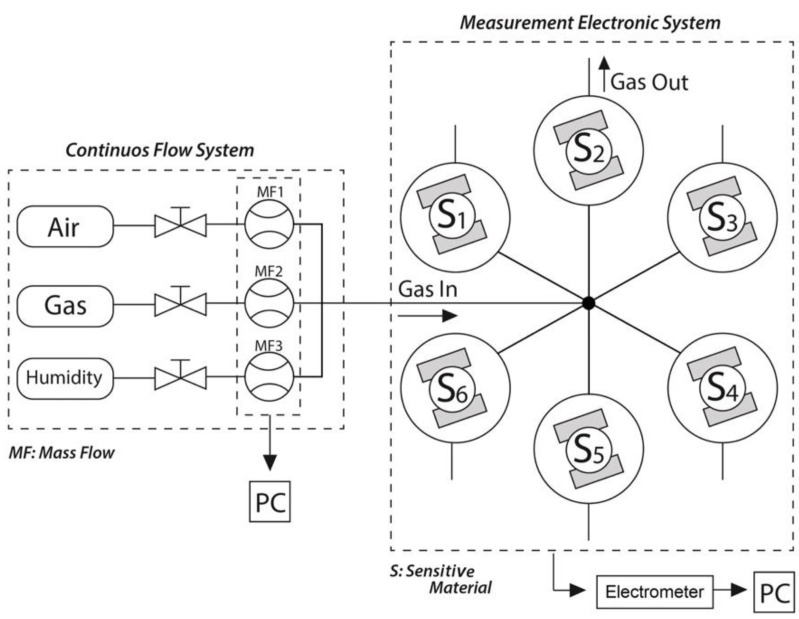
Schematic of the sensor test facility showing the gas lines passing through the mass flow controllers (MF) to the six sensors (S1–S6) characterized simultaneously.

The measurement of the DC resistance of the sensors was achieved with an electrometer (Keithley 6517B, Germering, Germany) with a multiplexer relay to simultaneously monitor several sensors (see the diagram of the test system in Figure 2, adapted from [59]). The sensor response was defined as R_a_/R_g_, where R_a_ and R_g_ are the resistance in dry/humid air and the resistance after exposure to the gas, respectively. The sensors were exposed to the target gas for a period of 5 min in the phase of the identification of the optimal temperatures and responses to gases. Subsequent tests (i.e., at the optimal temperature and with the selected gases) were performed by exposing the sensors to the target gases for 10 min. The sensors were tested over a period of 2 months, during which each sensor accumulated 400 h of operation under the different conditions (gases, temperatures, and humidities) used. The tests were performed in duplicate for each sensitive material to evaluate the repeatability of the sensors.

## 3. Results and Discussion

### 3.1. Optimum Loading of Solids in the Suspensions 

**Figure 3:**
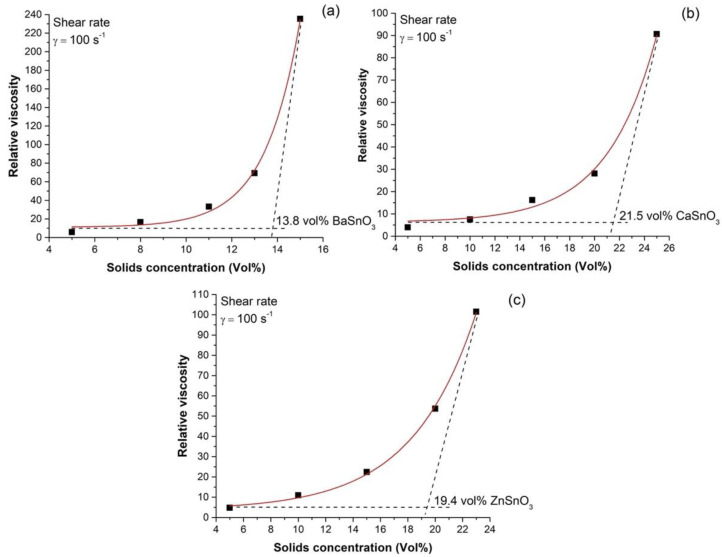
Relative viscosity $\left(\eta_{r}\right)$ as a function of solid content (φ) for the suspensions containing BaSnO_3_ (**a**), CaSnO_3_ (**b**), and ZnSnO_3_ (**c**) powders.

The amount of MSnO_3_ powders (M = Ba, Ca, Zn) to be used to form the suspensions was determined by graphs of viscosity (*η*) vs. volumetric fraction of solids (φ) (Figure 3). For the measurement of viscosity in the suspensions, a shear rate of 100 s^−1^ was used, a value commonly used in casting techniques. In this way, a value close to the maximum particle packing fraction was sought, in which a small increase in φ leads to a significant increase in viscosity. To find this value, in Figure 3, a tangent line to the curve of the relative viscosity increase was drawn in the region where $\eta_{r}\to \infty$ such that this line, intercepting the baseline (i.e., the minimum viscosity), allowed us to determine the values of the maximum optimal load (φ_m_) of solids used in this work. The volumes of solids were 13.7%, 20.8%, and 19.2% for the suspensions containing BaSnO_3_, CaSnO_3_, and ZnSnO_3_ powders, respectively. The suspensions with optimal filler content were poured into plaster moulds, as described in Section 2.2.

### 3.2. Densification and Porosity of Gas-Sensitive Materials 

**Figure 4:**
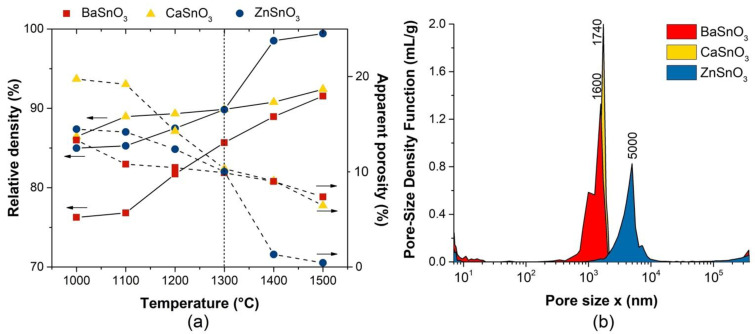
(**a**) Densification and apparent porosity, and (**b**) pore size distribution of the MSnO_3_ (M = Ba, Ca, Zn) bodies. The relative densities are related to the theoretical densities of 7.33 g/cm^3^ (BaSnO_3_), 5.53 g/cm^3^ (CaSnO_3_), and 6.79 g/cm^3^ (ZnSnO_3_).

Figure 4a shows the relative densities and the apparent porosity of the stannate ceramic bodies subjected to heat treatments at temperatures between 1000 and 1500 °C. The densification rates for the bodies were different and depended on the chemical nature of the stannate. The three systems achieved densifications greater than 75% from 1000 °C; the ZnSnO_3_ system obtained an appreciable increase in its relative density of ~90% from 1300 °C, and the densifications for the BaSnO_3_ and CaSnO_3_ systems were greater than 90% after 1400 °C. The apparent porosity, an indirect measure of the estimation of the degree of porosity of the sintered bodies, decreased with increasing temperature; at 1300 °C, the AP% presented values around 11% for the three systems. These results, as well as the degree of densification of the sintered bodies, allowed the selection of the appropriate temperature to thermally treat the shaped bodies that were used as sensitive materials. The selection criteria for this temperature were, on the one hand, to have densification values that would allow manual adaptation of the ceramic body to the electrode system shown in Figure 1b, and, on the other hand, to obtain an apparent porosity with similar values between samples and that favours the interaction between the sensor surface and the test gas. Therefore, the selected sintering temperature was 1300 °C for the three systems; at this temperature, the desired degrees of densification and values between 60–70% for the volume of permeable pore space (voids) were obtained. The volume of permeable pore space (voids) is a porosity value that only takes into account permeable pores, those that dry and then become wet when the sample is placed in water and boiling; other pores it does not recognize. Therefore, the effect of the porosities present in the sensitive materials was studied by mercury porosimetry and nitrogen absorption–desorption (Supplementary Materials). The pore size distribution curves obtained by mercury intrusion porosimetry show that all of the sensitive materials (MSnO_3_ (M = Ba, Ca, Zn)) sintered at 1300 °C presented a monomodal distribution, with average pore diameters ranging from 1600 to 5000 nm, as shown in Figure 4b. 

### 3.3. Structural, Compositional, and Morphological Studies

**Figure 5:**
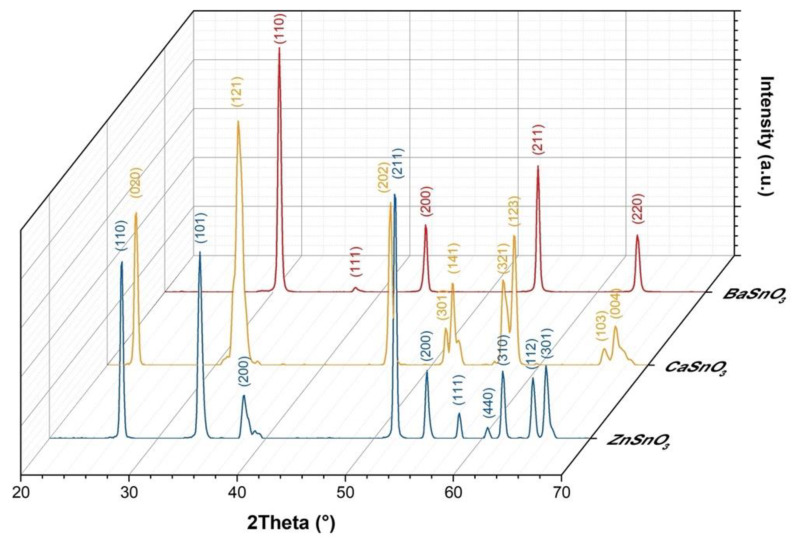
XRD patterns of sintered ceramic bodies based on MSnO_3_ perovskites (M = Ba, Ca, Zn).

Figure 5 shows the XRD patterns of the ceramic bodies sintered at 1300 °C manufactured from the suspensions of MSnO_3_ perovskite powders (M = Ba, Ca, Zn). The indexed diffraction peaks for each pattern correspond to BaSnO_3_, CaSnO_3_, and ZnSnO_3_, with JCPDS card Nos. 15-780, 31-312, and 28-1486, respectively. 

**Figure 6:**
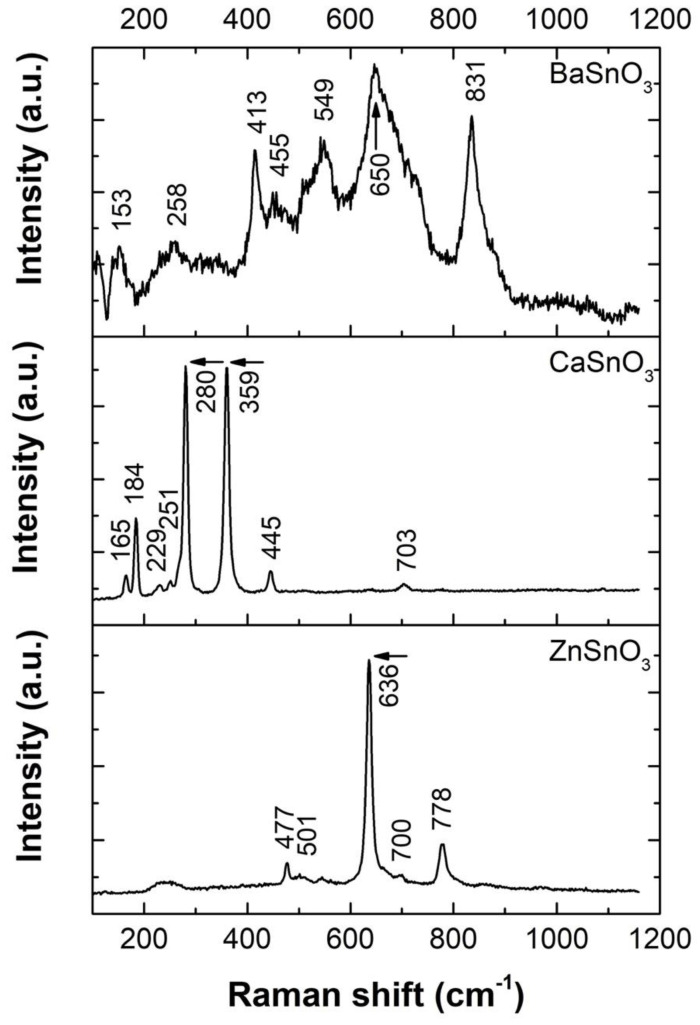
Room temperature (23 °C) Raman spectra of the sintered ceramic bodies based on MSnO_3_ perovskites (M = Ba, Ca, Zn).

The Raman spectra of the ceramic bodies of the MSnO_3_ system (M = Ba, Ca, Zn) measured at room temperature are shown in Figure 6. For the BaSnO_3_ sample, bands at 258, 413, 455, 549, 650, and 831 cm^−1^ are observed, similar to those identified in the study by James et al. [60]. According to the XRD pattern presented above, only the crystalline phase of BaSnO_3_ is observed, with an ideal cubic structure belonging to the space group Pm3m (O_h_). However, this should not show the first-order Raman active mode (this was predicted by group theory according to the irreducible representation 3F_1u_ (IR) + F_2u_ (silent) [60,61]). Therefore, the observed Raman bands can be assigned to the vibration modes of the SnO_6_ octahedron, which has O_h_ symmetry, in the distorted cubic structure of BaSnO_3_. The six fundamental vibrations of the SnO_6_ octahedron are the symmetrical stretching mode v_1_A_1g_, asymmetric stretching modes v_2_E_g_ and v_3_F_1u_, asymmetric bending mode v_4_F_1u_, symmetric bending mode v_5_F_2g_, and inactive mode v_6_F_2u_ [62]. In our study, the observed Raman activity can be attributed to defects that affect the translational periodicity of the crystal lattice in the sample, as well as oxygen vacancies.

In the Raman spectrum of the CaSnO_3_ sample, the bands located at 165, 184, 229, 251, 280, 359, 445, and 703 cm^−1^ are in agreement with the previous studies of Maul et al. [63] and Redfern et al. [64], in which a harmonic analysis was performed. According to the authors, the bands between 100 and 300 cm^−1^ have a higher contribution of Ca^2+^ and Sn^4+^ atoms and correspond to vibrations of Ca-SnO_3_ and O-Sn-O and a small contribution of apical O^2−^. For frequencies greater than 300 cm^−1^, the greatest contribution comes from oxygen ions, which are related to the torsion and stretching modes of SnO_3_ and Sn-O [65], respectively.

Finally, the Raman spectra corresponding to the ZnSnO_3_ sample show first-order vibrational active modes that agree well with the values reported at 477 cm^−1^ (E_g_), 636 cm^−1^ (A_1g_), and 778 cm^−1^ (B_2g_) for the structure SnO_2_ [66]. The most prominent Raman peak, located at 636 cm^−1^, was also observed by Mayedwa et al. [67]; this peak corresponds to the distinctive Raman shift of ZnSnO_3_ and is attributed to stretching vibrations of short M–O bonds, also coinciding with the XRD results reported in the same study with JCPDS card 28–1486. The weak bands at 501 and 700 cm^−1^ could correspond to infrared (IR) modes that can become weakly active when structural changes induced by disorder and size effects or even the presence of oxygen vacancies are introduced [68,69,70].

**Figure 7:**
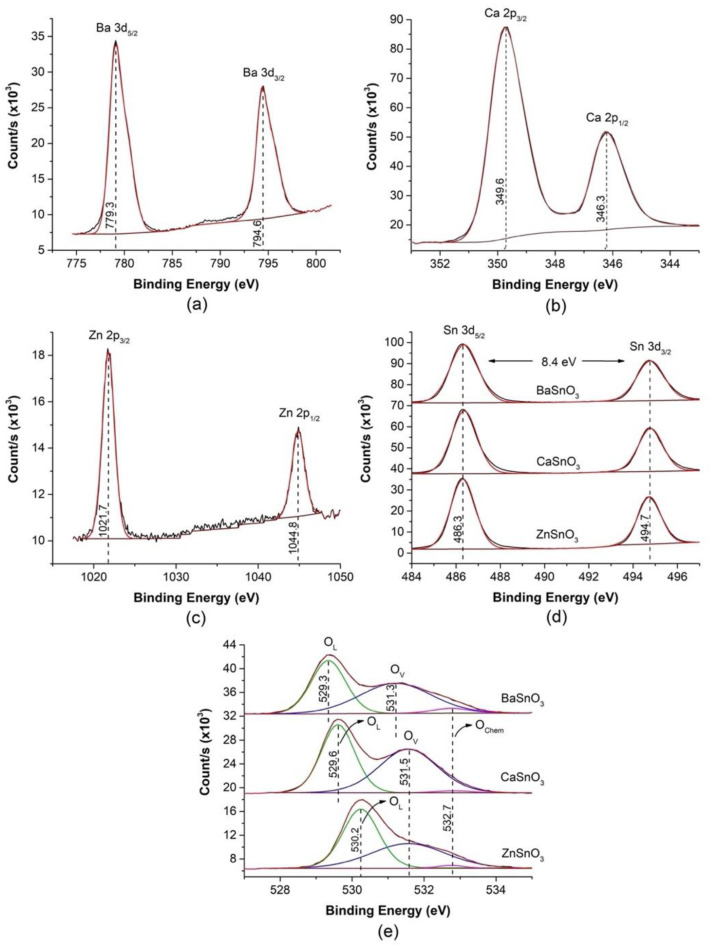
XPS spectra of the sintered ceramic bodies based on MSnO_3_ perovskites (M = Ba, Ca, Zn): (**a**) Ba-3d, (**b**) Ca-2p, (**c**) Zn-2p, (**d**) Sn -3d, and (**e**) O-1s (O_L_: lattice oxygen, O_V_: oxygen vacancies and O_Chem_: chemosorbed oxygen species).

The chemical composition (the valence states of metal ions) of the surface of the ceramic bodies of the MSnO_3_ system (M = Ba, Ca, Zn) was additionally investigated by XPS, as shown in Figure 7. 

The binding energies (BEs) obtained from the XPS spectra were calibrated by referring to the C 1s signal at 284.8 eV, corresponding to adventitious physisorbed carbon oxide. High-resolution XPS spectra of the Ba-3d, Ca-2p, Zn-2p, Sn-3d, and O-1s regions are shown in Figure 7. The Ba-3d_5/2_ and Ba-3d_3/2_ BEs in the BaSnO_3_ sample are located at 779.3 and 794.6 eV (Figure 7a), respectively, and separated by 15.3 eV, confirming the presence of Ba^2+^ species [71]. The states of Ca-2p_3/2_ and Ca-2p_1/2_ at 346.1 eV and 349.6 eV (Figure 7b), respectively, whose separation from each other is 3.5 eV, are attributed to Ca^2+^ [72,73] in the CaSnO_3_ sample. The bands at 1021.7 and 1044.8 eV, separated by 23.1 eV, are assigned to the Zn-2p_3/2_ and Zn-2p_1/2_ of the Zn^2+^ in ZnSnO_3_, respectively (Figure 7c) [74]. The spectra in Figure 7d reveal two peaks, at 486.3 and 494.7 eV with a separation of 8.4 eV, for the Sn-3d state in the stannates, which are attributed to Sn-3d_5/2_ and Sn-3d_3/2_, respectively. The maximum separation of Sn-3d in this study (8.4 eV) coincides with that observed for the Sn-3d reported for SnO_2_ [75,76,77]. The presence of Sn(II) can be ruled out due to the absence of subpeaks between the symmetrical peaks Sn-3d_3/2_ and Sn-3d_5/2_ as a result of deconvolution. The O-1s spectra (Figure 7d) show wide and asymmetric peaks and can be resolved into three fitting peaks in the 528–534 eV region, which has been observed in previous investigations of compounds with perovskite structures [30,31]. The first peak, at a lower BE of 529.3 eV in BaSnO_3_, 529.6 eV in CaSnO_3_, and 530.2 eV in ZnSnO_3_, is assigned to lattice oxygen (O_L_), and the other two components can be attributed to oxygen vacancies (O_V_) in the region between 531.2–531.5 eV and chemosorbed oxygen (O_Chem_) in the band at 532.7 eV. The results suggest a significant proportion of oxygen vacancies with respect to the lattice oxygen that is similar for all samples (1.17 O_V_/O_L_ for BaSnO_3_, 1.13 for CaSnO_3_, and 1.10 for ZnSnO_3_). These oxygen vacancy defects trap electrons from the conduction band of the perovskites to form abundant adsorbed oxygen ions and to favour gas-sensing performance [78].

**Figure 8:**
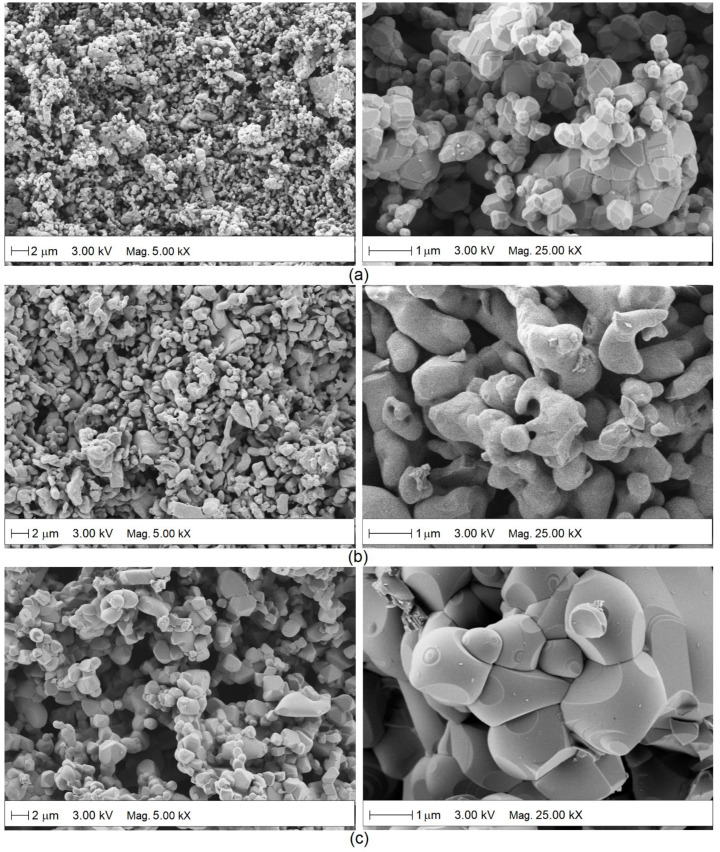
Surface morphology of the sensitive materials: (**a**) BaSnO_3_, (**b**) CaSnO_3_, and (**c**) ZnSnO_3_.

Figure 8 shows the SEM micrographs of the surface of the ceramic bodies formed with BaSnO_3_, CaSnO_3_, and ZnSnO_3_ powders sintered at 1300 °C. In the three samples, a high porosity is observed. The BaSnO_3_ sample has the smallest grain size (<500 nm) with a rhombohedral morphology (see Figure 8a). The largest grain size (~1 μm) was presented by the solids formed with CaSnO_3_ and ZnSnO_3_ powders, presenting spheroidal morphologies (Figure 8b,c). In addition, ZnSnO_3_ seems to be composed of large pores, which agrees with the results shown in Figure 4b.

### 3.4. Gas Sensing Properties

**Figure 9:**
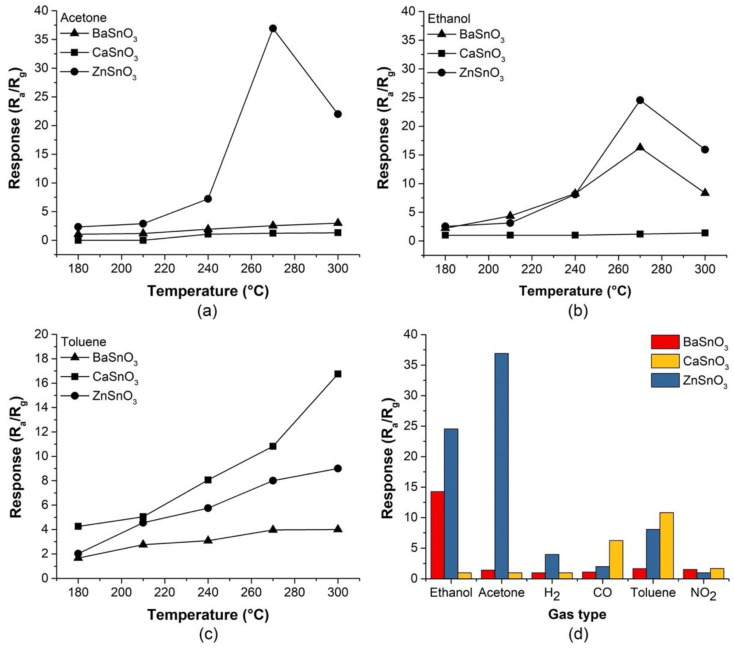
Response of the MSnO_3_ sensors (M = Ba, Ca, Zn) exposed for 5 min to 80 ppm of (**a**) acetone, (**b**) ethanol, and (**c**) toluene versus operating temperature. (**d**) Selectivity of the MSnO_3_ sensors (M = Ba, Ca, Zn) to various gases at 270 °C.

To investigate the sensing properties of the MSnO_3_ porous bodies (M = Ba, Ca, Zn), the optimal operating temperatures (T_op_) of the sensors were initially determined (i.e., the temperature at which the sensor response was highest). A temperature range of 180 to 300 °C was chosen to test the response of the sensitive materials to 80 ppm acetone, ethanol, and toluene. As a result of the tests, the average R_a_/R_g_ response of each porous body exposed to acetone is shown in Figure 9a. The response of the ZnSnO_3_ sensor increased appreciably, reaching a maximum at 270 °C, while the BaSnO_3_ and CaSnO_3_ sensors showed low response to temperature variation. When the samples were exposed to ethanol (Figure 9b), the BaSnO_3_ and ZnSnO_3_ sensors had a greater response with increasing temperature, reaching a maximum value at 270 °C. The CaSnO_3_ samples produced a weaker response than the BaSnO_3_ samples. In Figure 9c, with toluene gas, the sensors did not reach an optimal response in the range of working temperatures; however, the CaSnO_3_ sensor presented a stronger response than the other sensors, although temperatures higher than the range studied here were required. Figure 9a,b show that the response curves for the ZnSnO_3_ and BaSnO_3_ sensors exposed to acetone and ethanol, respectively, increased with increasing working temperature and then gradually decreased. The relative optimal working temperature (T_op_) can be explained by the cooperation of two opposite effects: (1) an increasing probability of activated detection reactions at low temperatures (e.g., for ZnSnO_3_ to acetone, between 180 and 270 °C) and (2) an increasing probability of adsorbed gas molecules to desorb before the detection reactions occur when the temperature is higher (e.g., for ZnSnO_3_ to acetone, over 220 °C) [79].

To test the selectivity of the sensors, they were exposed to fixed concentrations (80 ppm) of various volatile organic compounds, known as VOCs (acetone, ethanol, and toluene), among other gases (CO, H_2_, and NO_2_). The responses of the MSnO_3_ sensors (M = Ba, Ca, Zn) measured at T_op_ = 270 °C were compared in Figure 9d. It can be clearly observed that the ZnSnO_3_ sensor presented the strongest response in the presence of acetone gas. The BaSnO_3_ sensor responded very well to ethanol gas, although less than ZnSnO_3_; however, it suggested being more selective than ZnSnO_3_ due to the low responses obtained for the other gases. The CaSnO_3_ sensor, although it was not tested at its optimal temperature since that would be above 300 °C, showed a good response to toluene gas compared to the other VOCs.

**Figure 10:**
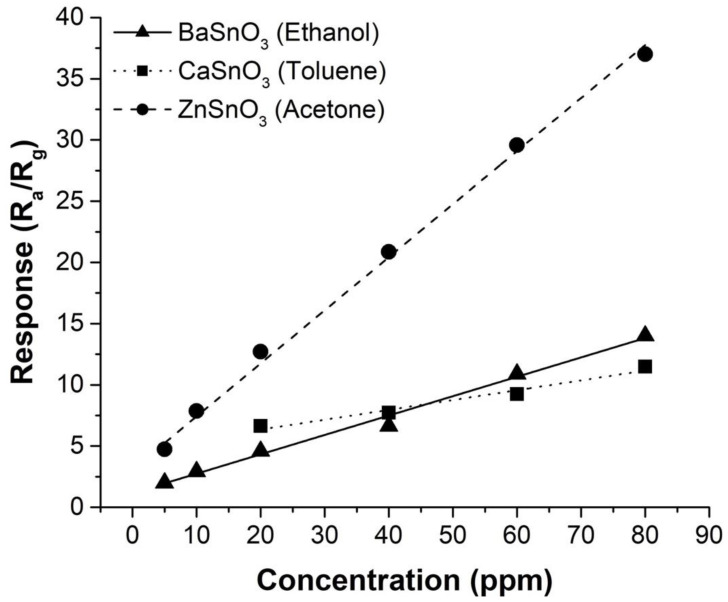
Fitting curve of the sensor responses of BaSnO_3,_ CaSnO_3_, and ZnSnO_3_ to ethanol, toluene, and acetone concentrations, respectively. The gas exposure time for each concentration was 5 min.

The responses of the sensors based on porous bodies of MSnO_3_ (M = Ba, Ca, Zn) showed superior gas detection of ethanol, toluene, and acetone. The typical curves of the response of the sensors to these gases as a function of the concentration are shown in Figure 10. There was a good linear relationship between the response and the concentration of tested gases. This linear fitting relationship provides an experimental basis for practical applications. Correspondingly, the sensitivity (i.e., the variation in the sensor response as a function of the change in the tested gas concentration from 5 to 80 ppm) showed higher values for ZnSnO_3_ to acetone (~40% ppm^−1^), BaSnO_3_ to ethanol (~16% ppm^−1^), and CaSnO_3_ to toluene (~8% ppm^−1^).

**Figure 11:**
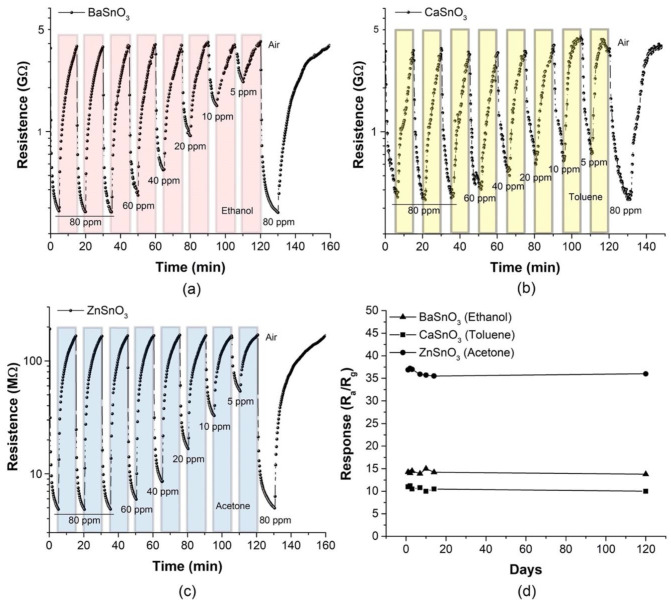
Dynamic response–recovery cycles of the (**a**) BaSnO_3_, (**b**) CaSnO_3_, and (**c**) ZnSnO_3_ sensors, exposed to different concentrations of ethanol, toluene, and acetone, respectively. (**d**) Long-term stability of the sensors (to 80 ppm and 270 °C).

Figure 11a–c shows the dynamics of the responses of the MSnO_3_ sensors (M = Ba, Ca, Zn) at the optimum operating temperature (270 °C) selected in this work. Initially, the responses were recorded by repeatedly exposing and purging to 80 ppm of the test gases (ethanol, toluene, or acetone) for three consecutive cycles. Then, the concentrations were varied from 60 to 5 ppm and again to 80 ppm. Reversible cycles were observed, in which the sensitive materials maintained their base electrical resistance (in air). Figure 11 also indicates the magnitude of the electrical resistance (plotted logarithmically), which was on the order of MΩ for the ZnSnO_3_ sensor and GΩ for the BaSnO_3_ and CaSnO_3_ sensors. These magnitudes can be influenced by the bulk and grain boundaries of the porous bodies [80,81]. It should be noted that the three perovskites showed a reduction in electrical resistance when in contact with reducing gases such as acetone, ethanol, and toluene, which indicates n-type semiconductor behaviour [27,28].

Considering the practical application of the sensors, the accuracy of long-term detection must be guaranteed. This stability was studied by measuring the average response of each sensor system exposed to the test gas at 80 ppm during the first 3 days and then at 7, 10, 14 and 120 days. As shown in Figure 11d, the long-term stability of the sensors had an almost constant response, which confirmed the high stability of sensors based on porous bodies of the MSnO_3_ system (M = Ba, Ca, Zn). The small variations in the response can be explained by the degree of passivation of the oxygen species adsorbed on the surface of each sensor, which over time can cause a difference in the resistance measured in air and the test gases (ethanol, toluene, and acetone).

**Figure 12:**
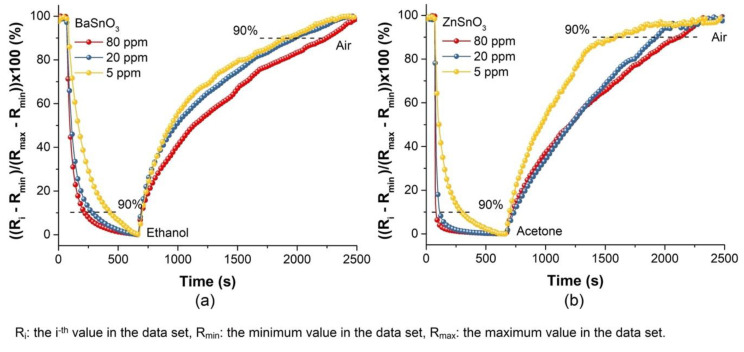
Response–recovery curves of the (**a**) BaSnO_3_ and (**b**) ZnSnO_3_ sensors exposed for 10 min to different gas concentrations of ethanol and acetone, respectively, at operating temperature of 270 °C.

Response time and recovery are important parameters of gas sensors and are considered when these devices are implemented in scenarios where real-time detection requires a “rapid response” according to the area of application. The response time (t_res_) was defined as the time required to reach 90% of the resistance difference after the injection of a test gas, and the recovery time (t_rec_) was defined as the time required for the sensor to recover 90% of its resistance in air. The characteristics of the dynamic response of the sensors based on BaSnO_3_ and ZnSnO_3_ porous bodies were investigated and showed a rapid response to ethanol and acetone gases, respectively, operating at 270 °C, as seen in Figure 12 (normalized data).

The values t_res_ and t_rec_ for the sensitive materials ZnSnO_3_ and BaSnO_3_ were calculated at 80, 20, and 5 ppm of the test gases at T_op_ = 270 °C, as indicated in Table 1. The results in Table 1 show that the ZnSnO_3_ sensor exposed to acetone gas exhibited faster responses (between 4 and 117 s) than the BaSnO_3_ sensor exposed to ethanol gas (between 72 and 219 s). A clear difference was observed in the response and recovery values, which suggested the importance of the porosity factor since the response and recovery were faster for the ZnSnO_3_ samples with larger pores; the open pores were effectively filled with chemisorbed species.

Table 2 summarizes the results of the main detection performance parameters of the porous bodies based on MSnO_3_ perovskites (M = Ba, Ca, Zn) compared with those corresponding to some previously published studies based on MSnO_3_ structures (M = Ba, Ca, Zn). The sensors obtained in this study showed results comparable to those of previous studies considering the gas concentrations used in each study. It is important to mention that the reports in Table 2 generally used more complex instrumentation in the elaboration of the sensor (e.g., physical vapor deposition (PVD) sputtering process) with respect to the method reported here or, in some cases, for the materials based on powders, did not carry out a control for the forming of the sensor film. The sensitivity of the ZnSnO_3_ samples prepared in this study was higher than those of the majority of previously published studies, even without incorporating second-phase materials such as Ag, Au, or SnO_2_, which are commonly used to enhance the sensitivity of intrinsic materials [11]. The ZnSnO_3_ samples in this study also showed a short response time, although the recovery time was longer than those in other studies. In the case of the BaSnO_3_ samples, the highest responses for similar ethanol concentrations could be connected to the higher operating temperatures (see Line 8, Table 2). It is also noted that the use of rare earth elements, such as La and Gd, in the works of Bhattacharya et al. [82,83] yielded significant improvements in the response and operating temperature of the BaSnO_3_ sensors compared to those of the samples prepared in this work. No reports were found on the detection of toluene vapours using sensors based on CaSnO_3_.

### 3.5. Gas Sensing Mechanism

The gas detection mechanism of stannates and many MOx semiconductors is controlled by the surface [89,90]. The most accepted model for explaining the sensitivity of semiconductors to gases postulates that the resistance changes are due to the species and the amount of chemisorbed oxygen on the surface. When sensors based on n-type semiconductors such as BaSnO_3_ and ZnSnO_3_ are exposed to air, the electrical resistance of the material is controlled by the concentration of adsorbed oxygen species (O^2−^, O^−^ or O^2−^). Previous studies suggest that for working temperatures below 100 °C, the majority of oxygen ions exist in the form of O^2−^; in the range of 100–300 °C, O^−^ ions are the stable oxygen species; beyond 300 °C, the dominant oxygen species is O^2−^ ions [86]. Generally, these oxygen species trap electrons and act as dispersion centres, effectively reducing the conductivity of the semiconductor. When the sensor was exposed to ethanol and acetone gases at temperature T_op_ (270 °C), these gases reacted with the adsorbed oxygen species, reducing their concentration, and thus increasing the conductivity of the semiconductor, i.e., reducing the electrical resistance, as observed in the measurements shown in Figure 11a–c. The occurring reactions can be explained as follows:

CH_3_CH_2_OH (gas) + 6O^−^ (ads) → 2CO_2_ + 3H_2_O + 6e^−^ (2)

CH_3_COCH_3_ (gas) + 8O^−^ (ads) → 3CO_2_ + 3H_2_O + 8e^−^ (3)

This indicates that the ethanol and acetone molecules are adsorbed on the surface of the porous body and react with the oxygen ions to produce CO_2_ and H_2_O. In this process, the electrons are released back to the conduction band, which results in a substantial increase in the density of charge carriers on the surface. This reduces the width of the semiconductor depletion layer and the potential barrier height [86], and as a consequence, the resistance of the sensitive material. This process is developed in the same way at the reaction sites of the remaining surface of the sensitive material, including the pores that are occupied by the diffusion of the gas until stabilizing. When the sensors are re-exposed to an air environment, the target gas is desorbed from the surface of the material, and the oxygen captures electrons from the conduction band to form oxygen ions, which increases the width of the electron depletion layer. The resistance of the sensing material returns to the initial value, as shown in Figure 11a–c.

In this work, the shaped bodies of BaSnO_3_ and ZnSnO_3_ presented good performance due to their porous structure, showing average pore sizes at both the macro- and nano-levels. In particular, the ZnSnO_3_ bodies were favourable because they had slightly larger macropores (5000 nm) than the BaSnO_3_ (1600 nm) and CaSnO_3_ (1740 nm) bodies, which would contribute to the diffusion of the gas in the bulk (volume) of the body. This difference in pore size was also observed, for example, in electron microscopy images of sections of the ZnSnO_3_ and BaSnO_3_ bodies, as seen in the Supplementary Materials (Figure S1). In addition, the ZnSnO_3_ samples also had pores with lower magnitudes (<10 nm in diameter), as did the BaSnO_3_ and CaSnO_3_ samples (Supplementary Materials Figure S2). Thus, the three bodies had a relatively similar surface area at the nanometric level. These characteristics, i.e., larger macropores and nanopores, also seemed to favour the response speed of the ZnSnO_3_ bodies (see Table 1).

The ZnSnO_3_ sensor also exhibited better responses than BaSnO_3_ and CaSnO_3_ in this study in terms of the operating temperature. Temperature plays an important role in the gas–solid interactions at the surface of sensitive materials. In this case, it was observed that the selected temperature (270 °C) for the tests with different gases and concentrations was closer to the optimal value of the ZnSnO_3_ sensor (for acetone and ethanol) than that of the BaSnO_3_ sensor (only for ethanol) and CaSnO_3_ (which seems to need higher thermal stimulation for acetone, ethanol, and toluene); see Figure 9. Hence, the ZnSnO_3_ sensors performed better for the tested VOCs.

### 3.6. The Influence of Humidity

**Figure 13:**
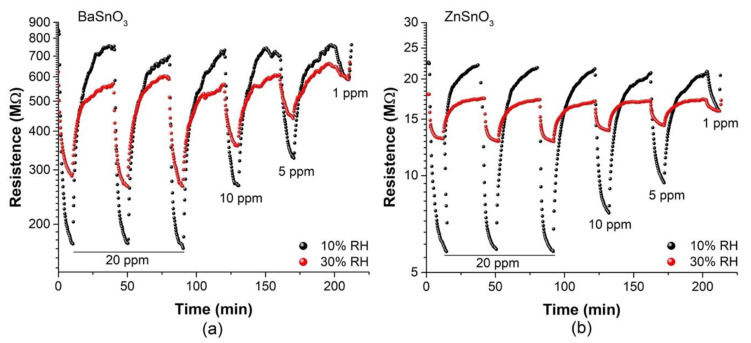
Dynamic response–recovery cycles in different relative humidity conditions. (**a**) BaSnO_3_ and (**b**) ZnSnO_3_ sensors, exposed for 10 min to various concentrations of ethanol and acetone, respectively, at operating temperature of 270 °C.

Currently, the development of MOx sensors for the detection of gases is of great interest due to increasing demands for environmental protection and their potential use in the diagnosis of diseases. Therefore, these sensors are used in humid environments due to the presence of water vapour in the environment. Considering that humidity is a negative factor for gas-sensing properties, we sought to confirm the influence of humidity on the sensitivity of the BaSnO_3_ and ZnSnO_3_ sensors towards ethanol and acetone vapours, respectively. Figure 13 shows the resistance changes (plotted logarithmically) for the BaSnO_3_ and ZnSnO_3_ sensors at different concentrations of ethanol and acetone, respectively, in an atmosphere with humidity (10 and 30% RH) at an operating temperature of 270 °C. Clearly, an increase in RH caused the resistance values of the sensors to decrease, especially in air, affecting the response and recovery rates. This occurred because the water molecules reacted with the chemisorbed oxygen species (O^−^) on the surface of the sensing material, which provided more electrons and reduced the reference resistance, resulting in a decrease in sensitivity [91]. This interaction in the ZnSnO_3_ and BaSnO_3_ sensors can be described by the following equations:

H_2_O + O^−^ (ads) + 2Ba → 2(Ba-OH) + e^−^ (4)

H_2_O + O^−^ (ads) + 2Zn → 2(Zn-OH) + e^−^ (5)

With respect to the previous observations and the consideration of the influence of OH species on the adsorption kinetics, we investigated the effect of humidity on the response and recovery rates of our sensors. Based on the responses of the sensors, the response and recovery times were calculated against different levels of RH with concentrations of 20 and 5 ppm of the target gases at 270 °C. Table 3 summarizes the results, indicating that under conditions of high humidity, the response takes longer because the water molecules adsorbed on the surface of the sensitive material can act as a barrier that prevents the adsorption of oxygen and gas molecules [92]. This results in a decrease in the active surface, reducing the sensitivity. In contrast, the recovery time was faster in humid than in dry conditions, specifically with BaSnO_3_ sensors. The faster recovery time may be due to an obstruction in the smallest pores by the OH molecules that interacted with the O^−^ species, causing the target gas molecules to be located mainly on the surface and not internally (in the bulk) and making their desorption faster and less difficult than when no humidity was introduced (Table 1).

## 4. Conclusions

In the present study, highly uniform porous bodies based on MSnO_3_ perovskites (M = Ba, Ca, Zn) were formed using the slip casting technique. Considering the structural advantages of the shaped bodies, the porous surfaces of the sensing materials had a large number of adsorption sites for gas molecules and were favourable for the diffusion of these molecules. The sensitivities of the BaSnO_3_, CaSnO_3_, and ZnSnO_3_ sensors to ethanol, toluene, and acetone were 16, 8, and 40% ppm^−1^, respectively, at an operating temperature of 270 °C, where the ZnSnO_3_ sensor exhibited the shortest response times (e.g., response time and recovery of 4 s and 1285 s, respectively, for 80 ppm acetone) as well as excellent medium-term stability (120 days). The improved gas detection properties of porous bodies, particularly ZnSnO_3_, with respect to other previously reported studies can be attributed to the porous structure, pores at both the macro (~5000 nm) and nano (<10 nm) levels, favouring the surface area and surface defects of the sensitive material.

## Figures and Tables

**Table 1:** Response (t_res_) and recovery (t_rec_) times of the BaSnO_3_ and ZnSnO_3_ sensors exposed to different gas concentrations of ethanol and acetone, respectively, at operating temperature of 270 °C. Results based on Figure 12.

Gas Concentration (ppm)	BaSnO_3_ (Ethanol)		ZnSO_3_ (Acetone)	
	t_res_ (s)	t_rec_ (s)	t_res_ (s)	t_rec_ (s)
80	72	1385	4	1285
20	98	1125	23	1159
5	219	1068	117	696

**Table 2:** Comparative table summarizing the results of this work and the sensing properties of various ZnSnO_3_ and BaSnO_3_ based sensors reported in the literature towards acetone and ethanol, respectively.

Sensing Material	Operating Temperature (°C)	Concentration (ppm)	Response (R_a_/R_g_)	Response/Recovery Time (s)	Year/Ref.
ZnSnO_3_ porous bodies	270	80	37	4/581	In this work
ZnSnO_3_ hollow polyhedrons with open nanoholes	240	50	12.48	17/10	2017/[84]
Silver-functionalized ZnSnO_3_ hollow nanocubes	280	100	30	2/3	2018/[85]
Au functionalized In-doped ZnSnO_3_ nanofibers	200	50	19.3	10/13	2019/[86]
Double-shell hollow SnO_2_/ZnSnO_3_ spheres	290	100	30	5/115	2021/[87]
BaSnO_3_ porous bodies	270	80	14.3	72/596	In this work
Nanocrystalline BaSnO_3_	300	20	~ 12.5	–	2015/[36]
Mesoporous BaSnO_3_ nanoparticles interconnected network	350	100	34.3	10/50	2017/[88]
La-doped BaSnO_3_	220	100	48	5/12	2020/[82]
Gd-doped BaSnO_3_	220	500	76	–	2020/[83]

**Table 3:** Response (t_res_) and recovery (t_rec_) time of the BaSnO_3_ and ZnSnO_3_ sensors exposed to different gas concentrations of ethanol and acetone, respectively, in humid atmosphere, at operating temperature of 270 °C. Results based on Figure 13.

Gas Concentration (ppm)	BaSnO_3_ (Ethanol)		ZnSO_3_ (Acetone)	
	t_res_ (s)	t_rec_ (s)	t_res_ (s)	t_rec_ (s)
	10/30% RH	10/30% RH	10/30% RH	10/30% RH
20	105/146	1065/971	45/52	1218/856
5	256/274	1003/873	146/158	1087/813

## Data Availability

Not applicable.

## Supplementary Materials

The following supporting information can be downloaded at: https://www.mdpi.com/article/10.3390/molecules27092889/s1. Figure S1: Scanning electron microscopy images and illustration of the gas sensing mechanism of the (a) BaSnO_3_ and (b) ZnSnO_3_ sensors; Figure S2: Pore size distributions by nitrogen absorption–desorption obtained by Quantachrome Instruments model NOVA 1000e for the BaSnO_3_, ZnSnO_3_, and CaSnO_3_ ceramic bodies.

## Sample Availability

Samples of the compounds are not available from the authors.
