# Supplementary material for: Gas Sensors Based on Porous Ceramic Bodies of MSnO3 Perovskites (M = Ba, Ca, Zn): Formation and Sensing Properties towards Ethanol, Acetone, and Toluene Vapours
Source: Molecules. 2022 Apr 30;27(9):2889. doi: 10.3390/molecules27092889 (PMC9105071; doi:10.3390/molecules27092889)
Supplement: Supplementary file 1 [file molecules-27-02889-s001.zip › molecules-1656429-SI.pdf]

Supplementary information

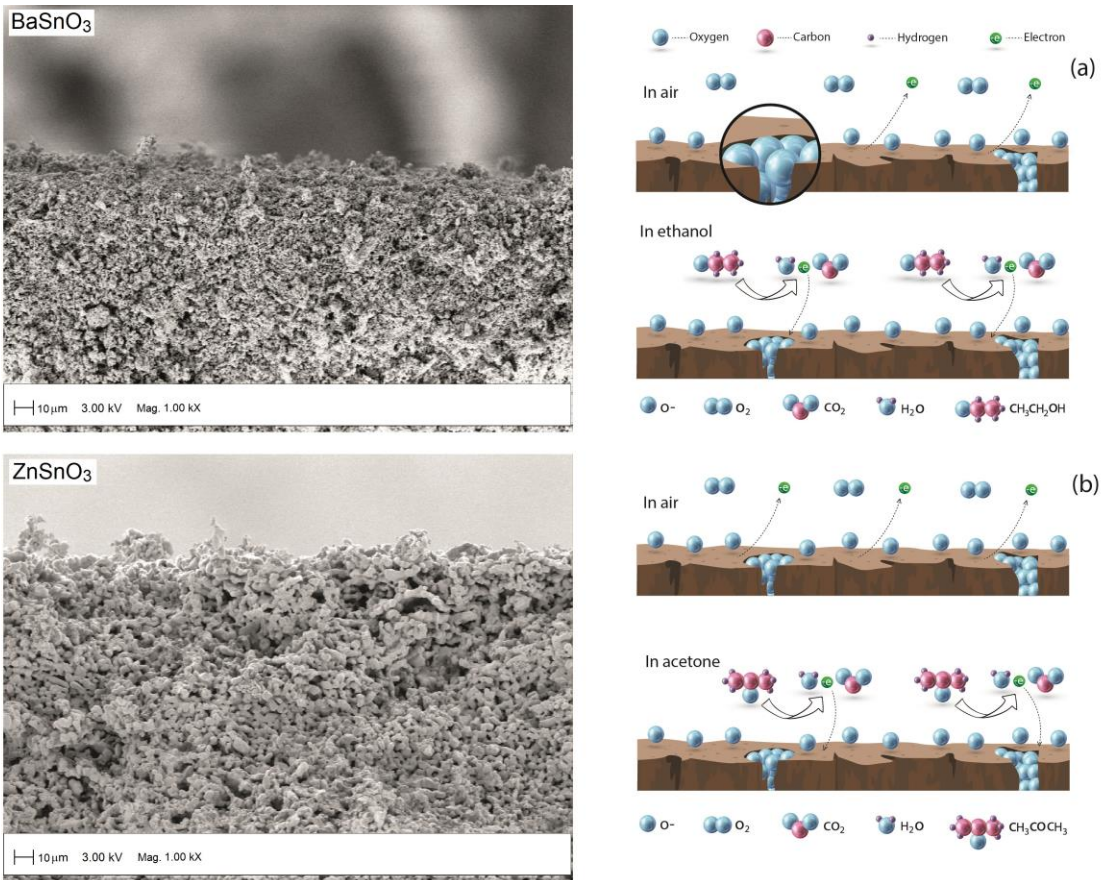

Fig. S1. Scanning electron microscopy images and illustration of the gas sensing mechanism of the (a) $BaSnO_3$ and (b) $ZnSnO_3$ sensors.

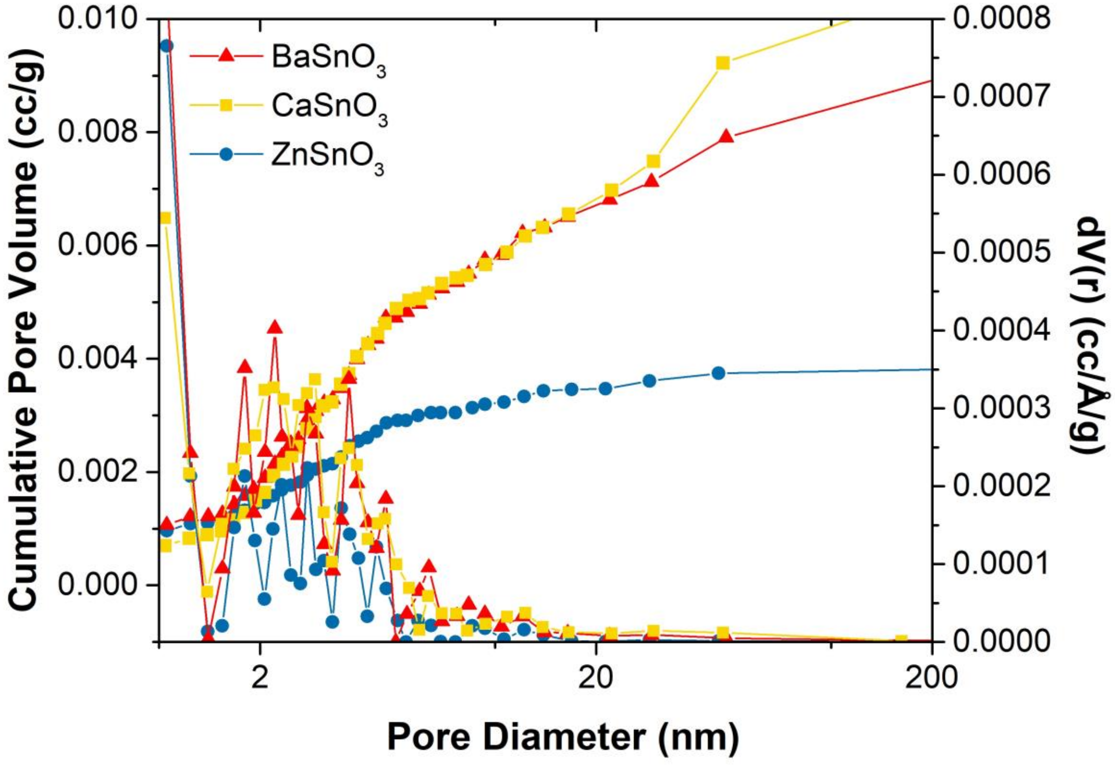

Fig. S2. Pore size distributions by nitrogen absorption-desorption obtained by Quantachrome Instruments modelo NOVA 1000e for the $BaSnO_3$, $ZnSnO_3$, and $CaSnO_3$ ceramic bodies.
